# Gene expression levels assessed by oligonucleotide microarray analysis and quantitative real-time RT-PCR – how well do they correlate?

**DOI:** 10.1186/1471-2164-6-59

**Published:** 2005-04-27

**Authors:** Peter B Dallas, Nicholas G Gottardo, Martin J Firth, Alex H Beesley, Katrin Hoffmann, Philippa A Terry, Joseph R Freitas, Joanne M Boag, Aaron J Cummings, Ursula R Kees

**Affiliations:** 1 Division of Children's Leukaemia and Cancer Research, Telethon Institute for Child Health Research and Centre for Child Health Research, The University of Western Australia, Perth, Australia; 2 Division of Biostatistics and Genetic Epidemiology, Telethon Institute for Child Health Research and Centre for Child Health Research, The University of Western Australia, Perth, Australia

## Abstract

**Background:**

The use of microarray technology to assess gene expression levels is now widespread in biology. The validation of microarray results using independent mRNA quantitation techniques remains a desirable element of any microarray experiment. To facilitate the comparison of microarray expression data between laboratories it is essential that validation methodologies be critically examined. We have assessed the correlation between expression scores obtained for 48 human genes using oligonucleotide microarrays and the expression levels for the same genes measured by quantitative real-time RT-PCR (qRT-PCR).

**Results:**

Correlations with qRT-PCR data were obtained using microarray data that were processed using robust multi-array analysis (RMA) and the MAS 5.0 algorithm. Our results indicate that when identical transcripts are targeted by the two methods, correlations between qRT-PCR and microarray data are generally strong (r = 0.89). However, we observed poor correlations between qRT-PCR and RMA or MAS 5.0 normalized microarray data for 13% or 16% of genes, respectively.

**Conclusion:**

These results highlight the complementarity of oligonucleotide microarray and qRT-PCR technologies for validation of gene expression measurements, while emphasizing the continuing requirement for caution in interpreting gene expression data.

## Background

The use of microarray technology to assess gene expression levels is now widespread in biology and, particularly in the clinical setting, the applicability of the methodology is likely to broaden as the technology evolves, data analysis procedures improve, and costs decline [1-3]. Two distinct microarray platforms, cDNA and oligonucleotide, are currently in general use [4]. While the relative merits of the two systems continue to be discussed [5], the validation of microarray results using independent mRNA quantitation techniques, including Northern blotting, ribonuclease protection, *in situ *hybridization, or quantitative real-time reverse transcription-polymerase chain reaction (qRT-PCR) remains a critical element of any microarray experiment [6,7]. Despite this, there have been few systematic validation studies of cDNA, or more noticeably, oligonucleotide microarray data using these independent approaches. For researchers to be confident with the interpretation of microarray results and for the establishment of consistent validation procedures in the microarray community for the purpose of data comparison, it is important that this issue be addressed.

We have undertaken an extensive series of experiments examining gene expression profiles in pediatric cancer specimens and normal tissues using oligonucleotide microarrays. For these studies, we used HG-U133A GeneChips (Affymetrix) which contain 22,283 probe sets representing approximately 14,500 human genes. To determine the preferred methodology for the analysis of our microarray data we compared the correlation between microarray expression scores obtained using two different data normalization procedures – Affymetrix MAS 5.0 [8], and robust multi-array analysis (RMA)[9] – with the expression levels obtained from follow-up verification experiments using qRT-PCR [10-12].

We found that the correlation between qRT-PCR and microarray expression data is generally strong. While our results highlight the complementarity of oligonucleotide microarray and qRT-PCR technologies for validation of gene expression measurements, the poor correlations that we observed for 13–16% of genes emphasizes the importance and continuing requirement for caution in interpreting gene expression data.

## Results

We have assessed the degree of correlation between microarray expression scores obtained for 48 genes using HG-U133A GeneChips with expression levels measured for the same genes using qRT-PCR. The genes that we assessed were identified as part of a larger study underway in the laboratory examining differential gene expression in pediatric leukemias and brain tumor specimens. The 48 genes were targeted for validation either on the basis of their differential expression between our subsets of interest (e.g. brain tumour vs normal brain specimens, leukemia specimens vs normal CD34+ stem cells) as determined by microarray analysis, or because they mapped to chromosomal regions of interest. In those cases where there were multiple microarray probe sets for particular genes, only data from those that showed evidence of differential expression were chosen for validation. For genes that were selected from chromosomal regions of interest and not necessarily on the basis of differential expression, correlations were carried out using data from the probe set deemed most specific for the gene of interest by the Affymetrix software (e.g. microarray probe sets designated *-at *are considered more specific than *-s-at *and *-x-at *probe sets).

**Figure 1:**
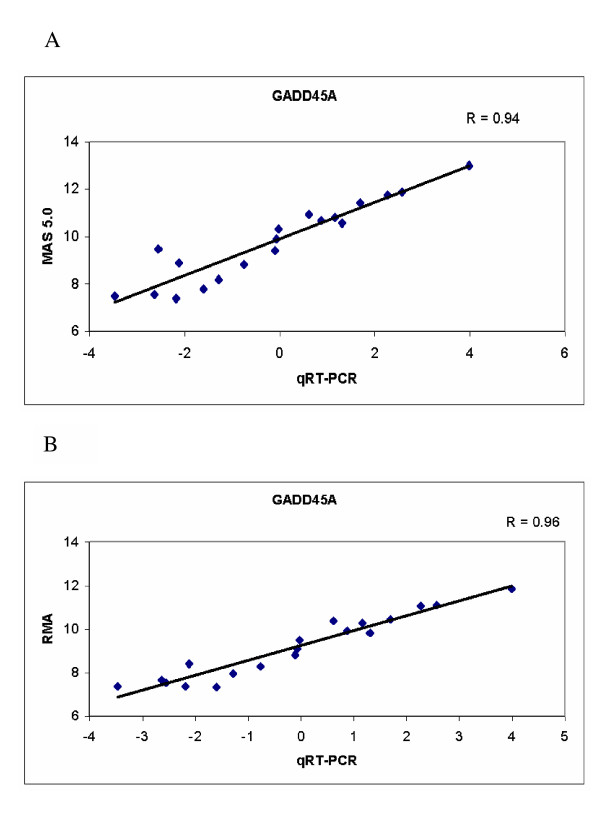
Examples of Pearson's correlations between gene expression levels determined by qRT-PCR and oligonucleotide microarray for one gene assessed in this study. The mRNA levels for the gene *GADD45A *were determined by qRT-PCR and correlated with microarray expression scores determined after data processing using MAS 5.0 software (A) or RMA (B). All data are shown as log_2_.

In total, 889 specimen/gene combinations were assayed by qRT-PCR and microarray in this study. Overall, statistically significant correlations (p < 0.05) were observed between qRT-PCR and RMA normalized data for 33/48 (69%) genes, and between qRT-PCR and MAS 5.0 normalized data for 32/48 (67%) genes (Tables 1 and 2, genes in bold). Typical data for a gene with a good correlation is presented in Figure 1. The correlation between the qRT-PCR data and microarray data normalized using either of the two methods was not significant (p > 0.05) for 14/48 (29%) genes (Tables 1 and 2, genes non-bold). Two genes, *FLJ20003 *and *RB*, showed significant correlations by RMA but not by MAS 5.0 analysis, while one gene, *GCLC*, had a significant correlation by MAS 5.0 but not by RMA.

**Table 1:** A comparison of average qRT-PCR, RMA, and MAS 5.0 scores and the corresponding correlation values for the 31 transcript-concordant genes assayed in this study for which the Affymetrix microarray probesets (Affy IDs) were deemed likely to recognize identical transcripts to qRT-PCR probes. Genes are ranked from lowest to highest average log_2 _RMA scores. Genes with significant correlations (p < 0.05) obtained by either normalization procedure are highlighted in bold. The number of specimens tested for each gene is included (n). Expression levels are shown as log_2_>.

**GENE**			**EXPRESSION**			**CORRELATION**	
		
**NAME**	**AFFY ID**	**n**	**RMA**	**MAS 5.0**	**qRT-PCR**	**RMA-qRT-PCR**	**MAS-qRT-PCR**
***LCE***	204256_at	22	4.79	7.01	0.27	**0.81**	**0.70**
***ALDH1A1***	212224_at	22	4.93	6.66	-2.93	**0.89**	**0.88**
***CFLAR***	211317_s_at	13	5.61	7.92	-0.12	**0.65**	**0.75**
***REL***	206036_s_at	13	5.63	8.36	0.53	**0.76**	**0.77**
***ABCC4***	203196_at	22	5.84	7.54	0.09	**0.78**	**0.89**
***FOXO1A***	202724_s_at	19	5.91	7.43	-2.05	**0.85**	**0.90**
***NOTCH2***	212377_s_at	13	6.19	8.24	-0.43	**0.77**	**0.82**
***TNFRSF21***	214581_x_at	13	6.22	8.03	1.98	**0.83**	**0.97**
***MADH9***	206320_s_at	19	6.24	5.47	-2.29	**0.87**	**0.74**
***PPM1D***	204566_at	30	6.29	8.80	0.50	**0.73**	**0.72**
***MAP7***	202889_x_at	22	6.42	6.27	-3.51	**0.85**	**0.87**
*DMBT1*	208250_s_at	19	6.49	7.25	-6.49	0.20	-0.11
***SNIP1***	219409_at	13	6.57	8.34	1.08	**0.69**	**0.77**
***OSF2***	210809_s_at	19	6.59	7.68	-1.26	**0.80**	**0.77**
***ATBF1***	208033_s_at	19	6.64	7.14	0.27	**0.81**	**0.84**
***KIT***	205051_s_at	22	6.70	7.51	-2.73	**0.86**	**0.87**
*P53*	201746_at	19	7.00	8.50	-3.44	0.41	0.11
***BAG3***	217911_s_at	19	7.04	8.61	-0.98	**0.79**	**0.82**
***RB***	203132_at	19	7.04	9.14	-2.82	**0.45**	0.38
***WBP4***	203599_s_at	19	7.28	8.97	-0.24	**0.62**	**0.74**
***BNIP2***	209308_s_at	13	7.58	9.79	0.56	**0.68**	**0.69**
*UMPCMPK*	217870_s_at	13	8.17	10.98	1.10	0.37	0.12
***DCAMKL1***	205399_at	19	8.18	9.23	-3.57	**0.76**	**0.89**
***OAZIN***	201772_at	30	8.22	10.36	-0.36	**0.72**	**0.77**
***LHFP***	218656_s_at	19	8.37	9.27	-0.46	**0.89**	**0.90**
***BTG3***	205548_s_at	13	8.47	10.54	0.83	**0.86**	**0.90**
***DCX***	204850_s_at	19	8.81	10.08	0.62	**0.87**	**0.88**
*TERF2*	203611_at	19	9.05	10.04	-0.14	0.32	0.31
***GADD45A***	203725_at	19	9.17	9.80	-0.12	**0.96**	**0.94**
***PRSS11***	201185_at	19	9.22	9.85	-3.54	**0.63**	**0.64**
***RAP1***	201174_s_at	19	10.34	11.59	-0.82	**0.83**	**0.84**

**Table 2:** A comparison of average qRT-PCR, RMA, and MAS 5.0 scores and the corresponding correlation values for the 17 genes assayed in this study for which the Affymetrix microarray probesets (Affy IDs) may not recognize the exact same transcript subsets recognized by qRT-PCR probes. Genes are ranked from lowest to highest average log_2 _RMA scores. Genes with significant correlations (p < 0.05) obtained by either normalization procedure are highlighted in bold. The number of specimens tested for each gene is included (n). Expression levels are shown as log_2_.

**GENE**			**EXPRESSION**			**CORRELATION**	
		
**NAME**	AFFY ID	**n**	**RMA**	**MAS 5.0**	**qRT-PCR**	**RMA-qRT-PCR**	**MAS-qRT-PCR**
*CDC14A*	210742_at	13	5.77	7.64	-0.67	0.31	0.26
*P125*	209175_at	19	6.61	8.43	0.54	0.11	-0.11
***GCLC***	202922_at	13	6.65	9.20	0.33	0.46	**0.56**
*MAP3K7*	206853_s_at	13	6.65	8.72	1.19	0.11	-0.10
*TIAL1*	202405_at	19	6.68	7.86	0.30	0.32	0.17
***FLJ20003***	219067_s_at	19	6.71	8.54	0.64	**0.64**	0.34
*RUNX1*	210365_at	13	6.95	9.24	1.47	0.29	0.28
*PLEKHA1*	219024_at	19	6.99	8.18	-2.88	-0.40	-0.28
*FLJ12661*	218420_s_at	19	7.35	8.50	0.57	-0.08	-0.17
***RGC32***	218723_s_at	19	7.36	8.11	-3.20	**0.85**	**0.96**
*WDR11*	218090_s_at	19	7.96	9.04	0.60	0.12	0.01
***RFC3***	204127_at	19	8.10	9.77	1.20	**0.62**	**0.64**
*ASAH1*	213702_x_at	22	8.30	10.29	1.46	0.29	0.27
***P38IP***	220408_x_at	19	8.35	9.55	0.76	**0.73**	**0.65**
***BUB3***	201456_s_at	19	8.41	9.70	0.60	**0.64**	**0.61**
*SAC2*	203607_at	19	8.86	10.21	0.35	0.22	0.12
***TSC22***	215111_s_at	19	10.56	11.87	-1.34	**0.83**	**0.82**

By careful analysis of the relevant databases (see Methods) we identified a subset of 31 genes for which the microarray probe-sets were deemed to recognize the exact same transcript or subset of transcripts as the qRT-PCR probes (Table 1). When we assessed the levels of correlation for this group of 31 transcript-concordant genes a higher proportion of significantly correlating scores was observed; 84% (26/31) for MAS 5.0 normalized data and 87% (27/31) for RMA normalized data (Table 1, genes in bold). In addition, the average correlations between the MAS 5.0 or RMA data and the qRT-PCR data for this subset of genes were very similar (0.71 and 0.72, respectively). In contrast, for the remaining 17 genes for which the Affymetrix microarray probe-sets may not recognize the same subset of transcript(s) recognized by qRT-PCR probes, significant correlations were observed for only 41% (7/17) genes by either MAS 5.0 and RMA (Table 2). All genes with poor correlations were tested on the same numbers of samples as those genes that did correlate, and there was no relationship between sample type and whether or not correlation was significant. Separate genes were targeted for each sample type. Using a two sample t-test, the average correlations between RMA-qRT-PCR scores and MAS-qRT-PCR scores for the transcript concordant genes in Table 1 were significantly higher than the average of the equivalent correlations for the non-concordant genes in Table 2 (RMA-qRT-PCR Table 1 vs 2, p = 0.0005; MAS-qRT-PCR Table 1 vs 2, p = 0.0003).

**Figure 2:**
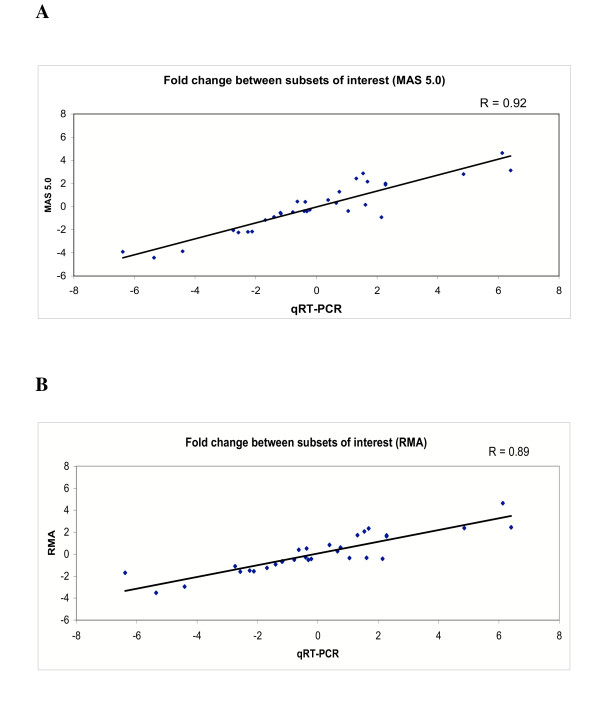
Pearson's correlations between fold-change in average gene expression levels between subsets of interest assessed by qRT-PCR and either MAS 5.0 software (A) or RMA (B) for the 31 transcript-concordant genes (see Table 1). All data are shown as log_2_.

Determining fold-changes in gene expression levels between subsets of interest is often a major aim of microarray studies. To address this issue, we analyzed fold-change in average gene expression levels between our subsets of interest (e. g. tumor vs normal) by both qRT-PCR and RMA or MAS 5.0 microarray scores for the same genes. Only the 31 transcript-concordant genes were considered in this analysis (Table 1). From a total of 587 specimen/gene combinations we found a significant and strong correlation in mean fold-change using both RMA (r = 0.89, p < 0.05) and MAS 5.0 (r = 0.92, p < 0.05) (Figure 2a, b). Interestingly, we noticed a trend towards poorer correlation for genes that exhibited fold-change differences of <1.5 between subsets of interest based on microarray expression scores compared to those with fold-change differences of >1.5 (data not shown). The slopes of the two regression lines in Fig. 2 are significantly greater than one [RMA vs qRT-PCR = 1.49 (95%CI = 1.20, 1.77); MAS vs qRT-PCR = 1.23 (95% CI = 1.03, 1.42)].

## Discussion

Microarray expression analysis has revolutionized many facets of biology and will continue to be applied widely. However, significant questions remain with regard to the generation, analysis, and in particular, interpretation of microarray data. Although the validation of microarray expression results obtained for specific genes using independent techniques is still considered a desirable component of any microarray experiment, the genes selected for validation *a priori*, are usually identified from the microarray data. The selection is based on the implicit assumption that there is a good correlation between the microarray data and actual mRNA levels in the cells under investigation. One fundamental issue that has not been adequately addressed is how well microarray expression scores reflect actual mRNA levels in the sample being examined.

To facilitate data comparison between research groups it is important that the microarray community moves to adopt consistent validation methodologies. This is especially important if microarray technology is to play a role in the clinical setting [13]. However, the choice of validation methodology remains a contentious issue [14]. To date, qRT-PCR is the method of validation that has been used in the majority of published microarray studies, presumably because it is a rapid, sensitive, high throughput procedure that requires minimal amounts of test material compared to techniques such as Northern blotting or ribonuclease protection assays. As is the case for many studies, including ours, qRT-PCR is often the only feasible approach when rare or unique tissues are investigated. For these reasons, it would appear likely that qRT-PCR will continue to be used extensively for the validation of microarray expression data [15]. To our knowledge, this study is the most extensive and practical examination of mammalian cells that focuses on the degree of correlation between expression level measurements obtained by oligonucleotide microarray analysis and qRT-PCR.

We observed strong correlations (p < 0.05) for the majority (>87%) of the 31 transcript-concordant genes that we examined in this study. In addition, although the MAS 5.0 software and RMA use different algorithms for the normalization of microarray data [8,9] we found that the degree of correlation between microarray and qRT-PCR results was very similar irrespective of the normalization procedure employed.

Our data clearly demonstrate that similar microarray scores for different genes do not necessarily mean that similar qRT-PCR scores will be obtained. For example, *ATBF1, OSF2*, and *SNIP1 *yielded similar average log_2 _RMA scores (~6.6) but the average log_2 _qRT-PCR scores for the same genes were substantially different (0.27, -1.26, and 1.08, respectively). Similarly, *KIT *and *ABCC4 *exhibited identical average log_2 _MAS 5.0 scores (~7.5), while the corresponding average log_2 _qRT-PCR scores were -2.73 and 0.09, respectively. The finding that genes with similar microarray expression scores were unlikely to have similar qRT-PCR results presumably reflects the different hybridization kinetics of the probe sets for each gene. This observation has the major implication that on the basis of the qRT-PCR data that we obtained, it is generally not feasible to predict the true expression level of one gene based on the microarray expression score of another. In addition, we observed significant correlations for many genes with microarray expression scores, at least by RMA, of less than 100 (~log_2_100 = 6.64), which is at the lower end of the range of microarray scores we obtained in this study (range 6–23000). This finding indicates that the exclusion of genes with low microarray expression scores (e.g. <100) from further analysis, as has been adopted by some research groups in early microarray studies, may not be justified.

Determining fold-changes in gene expression levels between subsets of interest is often a critical aim of microarray studies. We found a significant and strong correlation using RMA (r = 0.89, p < 0.05) and MAS 5.0 (r = 0.92, p < 0.05). These data indicate that the direction of change of gene expression levels (i.e. either up or down regulation) between subsets of interest is accurately predicted by comparison of average microarray expression scores. Again, the fold-change correlations we observed were very similar irrespective of the normalization procedure we employed. Consistent with the results of Yuen et al (2001)[16], fold change results determined by qRT-PCR were significantly greater than fold change assessed for the same genes by microarray analysis.

A recent study addressing gene expression profiles in *Arabidopsis *reported a good correlation between oligonucleotide microarray and SYBR green qRT-PCR data when ratios of gene expression in shoot tissue versus root tissue were compared for highly expressed genes. However, the correlations between shoot versus root ratios were generally poor for genes expressed at low levels [17]. We observed a similar trend towards poorer correlation for genes that exhibited fold-change differences of <1.5 between subsets of interest based on microarray expression scores compared to those with fold-change differences of >1.5. It is likely that this trend relates to the fact that small variations in mRNA levels (<2-fold) can be accurately detected by qRT-PCR, while the smaller dynamic range of microarrays means that the same changes may not be accurately reflected by microarray expression scores, especially for genes expressed at low levels (<1.5 pM or approximately 3.5 copies/cell) [18,19]. This latter point is a likely explanation for the poor correlation observed for one gene, *DMBT1*, which is expressed at very low levels according to our qRT-PCR data. Etienne *et al*., 2004 [20] observed a lower overall correlation between microarrray and semi-quantitative RT-PCR data compared to our study. These authors hypothesized that in addition to genes with low expression levels, those with very high expression levels or a greater percentage of absent calls, may show lower levels of correlation between Affymetrix expression scores and semi-quantitative RT-PCR data. We considered these issues in relation to the other poorly correlating genes in our study and found that none were expressed at levels that approach the fluorescence ceiling for the Affymetrix scanner (~50000). In addition, the absolute number or percentage of absent calls did not correlate significantly (p > 0.05) with the level of correlation between qRT-PCR results and microarray data (data not shown). It is possible that the differences between our results and those of Etienne and co-workers are related to the particular semi-quantitative RT-PCR methodology employed by these researchers, which may not be as sensitive as qRT-PCR, and as the authors point out, may not detect certain low level transcripts.

In addition to *DMBT1 *mentioned above, we identified 13 other poorly correlating genes from the 48 genes we assessed. Careful analysis of the alternative transcript data available through the LocusLink database  indicated that for 10 of these 13 genes, different subsets of alternative transcripts may be recognized by microarray probe sets and qRT-PCR probes. Hence, this may be the explanation for the poor correlations observed for these genes. Possible explanations for the poor correlations that were observed for the three remaining genes (*p53, UMPCMPK*, and *TERF2*), all of which were transcript-concordant, include the existence of alternative cross-hybridising transcripts differentially recognized by the oligonucleotide probe sets and qRT-PCR probes, gene specific variation related to the different hybridization kinetics associated with the two technologies, and misleading results associated with errors in GenBank sequence data and/or probe set annotations [21]. Additional experimental data will be required to address these possibilities. It is important to note that in our hands the reproducibility of both the qRT-PCR and oligonucleotide microarray methods is very high [22,23]. Hence, it is unlikely that poor correlations observed in our study are associated with issues of experimental precision.

Interestingly, the microarray and qRT-PCR expression data correlated well for five genes for which the microarray probe sets were deemed unlikely to recognize the same transcripts as the qRT-PCR probes. These data suggest that despite the possibility of differential transcript recognition, identical transcripts were being detected by both assays in the particular tissues involved.

## Conclusion

Our data indicate that correlations between qRT-PCR and microarray data are generally strong; a result that is particularly encouraging for those researchers with access to only very limited amounts of rare or unique test specimens. Our data also emphasize the importance of ensuring that qRT-PCR probes recognize the same transcript(s) as the microarray probe set. Finally, the 13–16% non-concordance that we observed indicates that independent validation of expression data continues to be an important consideration.

## Methods

### Specimens

Informed consent for the use of tissues for research purposes was obtained for all individuals involved in this study according to hospital and Australian National Health and Medical Research (NHMRC) guidelines.

We extracted total RNA from 64 specimens, including 13 primary pediatric brain tumors, six pediatric brain tumor cell lines, two normal adult brain cortices, and one fetal brain germinal matrix. We also obtained total RNA from fetal brain pooled from multiple individuals (Clontech). In addition, total RNA was extracted from 36 pediatric acute lymphoblastic leukemia bone marrow specimens and from CD34^+ ^hematopoietic stem cells isolated from the bone marrows of 5 normal individuals. Ficoll-hypaque purified leukemia cells or cryopreserved bone marrow specimens were snap frozen and stored in liquid nitrogen until required. Total RNA was extracted from ~1 × 10^6 ^– 2 × 10^7 ^live cells. Primary brain tumour specimens (10 – 150 mg) were either wrapped in foil or placed in RNAlater (Ambion) immediately after resection and stored at -80°C. Brain tumour cell lines were processed directly from tissue culture.

### RNA extraction, preparation of target cRNA and hybridization to HG-U133A GeneChips

Total RNA was extracted from all specimens using a combination of TRIZOL reagent (Invitrogen), RNeasy Mini kit (Qiagen) and ethanol precipitation. Following the TRIZOL reagent procedure, 0.53 volumes of 100% ethanol were added drop-wise to the aqueous phase and the mixture applied to RNeasy mini columns according to the manufacturer's instructions. Further purification and concentration was achieved through an additional ethanol precipitation. The integrity of the RNA preparation was assessed using agarose gel electrophoresis and analysis on an Agilent 2100 Bioanalyzer (Agilent Technologies). Biotinylated cRNAs for hybridization were prepared from total RNA according to Affymetrix protocols. Agarose gel electrophoresis was used to confirm the integrity of labelled cRNA and to assess its fragmentation products. Biotinylated cRNA preparations (15 μg) were hybridized to HG-U133A arrays, which were subsequently washed, stained, and scanned using a GeneArray Scanner (Agilent Technologies) according to the Affymetrix protocol.

### Processing and statistical analysis of microarray data

Array images were reduced to intensity values for each probe (*cel *files) using Affymetrix MAS 5.0 software and only those microarrays meeting acceptable Affymetrix quality control criteria were considered for further analysis. *Cel *files were then processed using either the MAS 5.0 software [8] or RMA (Bioconductor release 1.2) [9], an alternative algorithm that is publicly available at . The MAS 5.0 algorithm uses a scalar normalization technique taking into account perfect match (PM) and mismatch (MM) probe pairs to correct for non-specific hybridization, while RMA is based on a quantile normalization approach which ignores MM values. All microarrays processed using the MAS 5.0 software were scaled to a standard target intensity of 500. For comparison purposes, all microarray and qRT-PCR data are presented as log_2 _and absent/present calls generated by the MAS 5.0 software were not taken into account.

Pearson's correlations were used for the comparison of qRT-PCR and microarray data and p-values were obtained using Fisher's z-transformation. Correlations were considered significant at p < 0.05.

### Bioinformatics

To determine whether transcripts recognized by microarray probe sets [24] were likely to be identical to those detected by qRT-PCR probes, alternative splicing patterns for each gene were thoroughly reviewed using LocusLink  and Ensembl . Any full-length human mRNA or cDNA sequences demonstrating alternative splicing, in addition to NCBI-reviewed Reference Sequences (RefSeq), were considered as potential isoforms for each gene. Using BLAST alignments  of probe and cDNA sequences, the members of each isoform 'family' that could be targeted by either qRT-PCR or microarray were identified (typically multiple isoforms for each gene). The potential number of isoforms recognised by each technology were then compared. Probes which targeted exactly the same isoform subsets for each gene were considered 'transcript-concordant' and placed in Table 1; those for which at least one of the targeted isoforms differed (regardless of the number of matching isoforms) were considered 'non transcript-concordant' and placed into Table 2.

### qRT-PCR

All qRT-PCR assays were carried out using primer and probe sets from Applied Biosystems (ABI Assays on Demand, ). Each assay was designed using ABI's primer/probe selection algorithm and bionformatics pipeline which includes access to both public and Celera DNA sequence databases. The combination of gene specific primers and a gene specific probe ensures a high degree of specificity.

Aliquots of total RNA extracted for microarray analysis as described above were used for qRT-PCR experiments according to the manufacturer's protocols (ABI). All ABI Assays on Demand are designed to generate amplicons of 50–150 bp and are carried out using identical cycling conditions. 1–2 ug total RNA (quantitated by spectrophotometer at OD_260_) was used for each RT reaction. Three RT reactions were pooled and all qRT-PCR reactions were carried out using aliquots from the pool. We did not detect DNA contamination in any of our total RNA preparations after qualitative assessment using an Agilent Bioanalyzer. All qRT-PCR assays for a particular gene were undertaken at the same time under identical conditions and carried out in duplicate. All qRT-PCR experiments were run on an ABI 7700 sequence detector.

For all qRT-PCR assays the expression levels of target genes were normalised to the levels of the *ACTB *housekeeping gene utilising a standard curve method for quantitation as described previously [25]. Serial dilutions of cDNAs generated from selected cell lines that expressed target genes at a suitable level were used to generate a standard curve for each target gene and *ACTB*. The standard curves were then used to determine expression values (expressed as ng cDNA template) for each target gene after qRT-PCR analysis of each test specimen. Relative expression values for each target gene were expressed as a ratio of target gene expression level to *ACTB *expression level in the same specimen. These ratios were then correlated with the microarray data.

## Authors' contributions

PBD and NGG contributed equally to this work and were responsible for designing the study, analysing, collating, and interpreting the data, and preparing the manuscript. MJF carried out the statistical analysis, AHB and KF assisted with data analysis, experimental design, and data interpretation. PAT, JRF, JMB, AJC and NGG carried out the microarray and qRT-PCR experiments. URK supervised all aspects of the study and preparation of the manuscript.
